# Isolation of Mitochondria From Yeast to Estimate Mitochondrial Pools of Inorganic Phosphate

**DOI:** 10.21769/BioProtoc.5370

**Published:** 2025-07-05

**Authors:** Swagata Adhikary, Vineeth Vengayil, Sunil Laxman

**Affiliations:** 1 Institute for Stem Cell Science and Regenerative Medicine (BRIC inStem), Bengaluru, India; 2 Manipal Academy of Higher Education, Manipal, India

**Keywords:** Mitochondria isolation, Inorganic phosphate, Differential centrifugation, Immunoprecipitation, *Saccharomyces cerevisiae*

## Abstract

Mitochondria are dynamic organelles with essential roles in energetics and metabolism. Several metabolites are common to both the cytosolic and mitochondrial fractions of the cell. The compartmentalization of metabolites within the mitochondria allows specialized uses for mitochondrial metabolism. Inorganic phosphate (Pi) is one such critical metabolite required for ATP synthesis, via glycolysis and mitochondrial oxidative phosphorylation. Estimating total cellular Pi levels cannot distinguish the distribution of Pi pools across different cellular compartments, such as the cytosol and mitochondria, and therefore separate the contributions made toward glycolysis or other cytosolic metabolic processes vs. mitochondrial outputs. Quantifying Pi pools in mitochondria can therefore be very useful toward understanding mitochondrial metabolism and phosphate homeostasis. Here, we describe a protocol for the fairly rapid, efficient isolation of mitochondria from *Saccharomyces cerevisiae* by immunoprecipitation for quantitative estimation of mitochondrial and cytosolic Pi pools. This method utilizes magnetic beads to capture FLAG-tagged mitochondria (Tom20-FLAG) from homogenized cell lysates. This method provides a valuable tool to investigate changes in mitochondrial phosphate dynamics. Additionally, this protocol can be coupled with LC–MS approaches to quantitatively estimate mitochondrial metabolites and proteins and can be similarly used to assess other metabolite pools that are partitioned between the cytosol and mitochondria.

Key features

• This protocol describes how to isolate mitochondria from *Saccharomyces cerevisiae* for quantitative estimation of inorganic phosphate or other metabolites.

• Mitochondria are efficiently isolated by immunoprecipitation using magnetic beads, bypassing the need for time-consuming density-based centrifugation.

• This method can be integrated into LC–MS-based workflows to quantify mitochondrial metabolites and proteins.

## Graphical overview


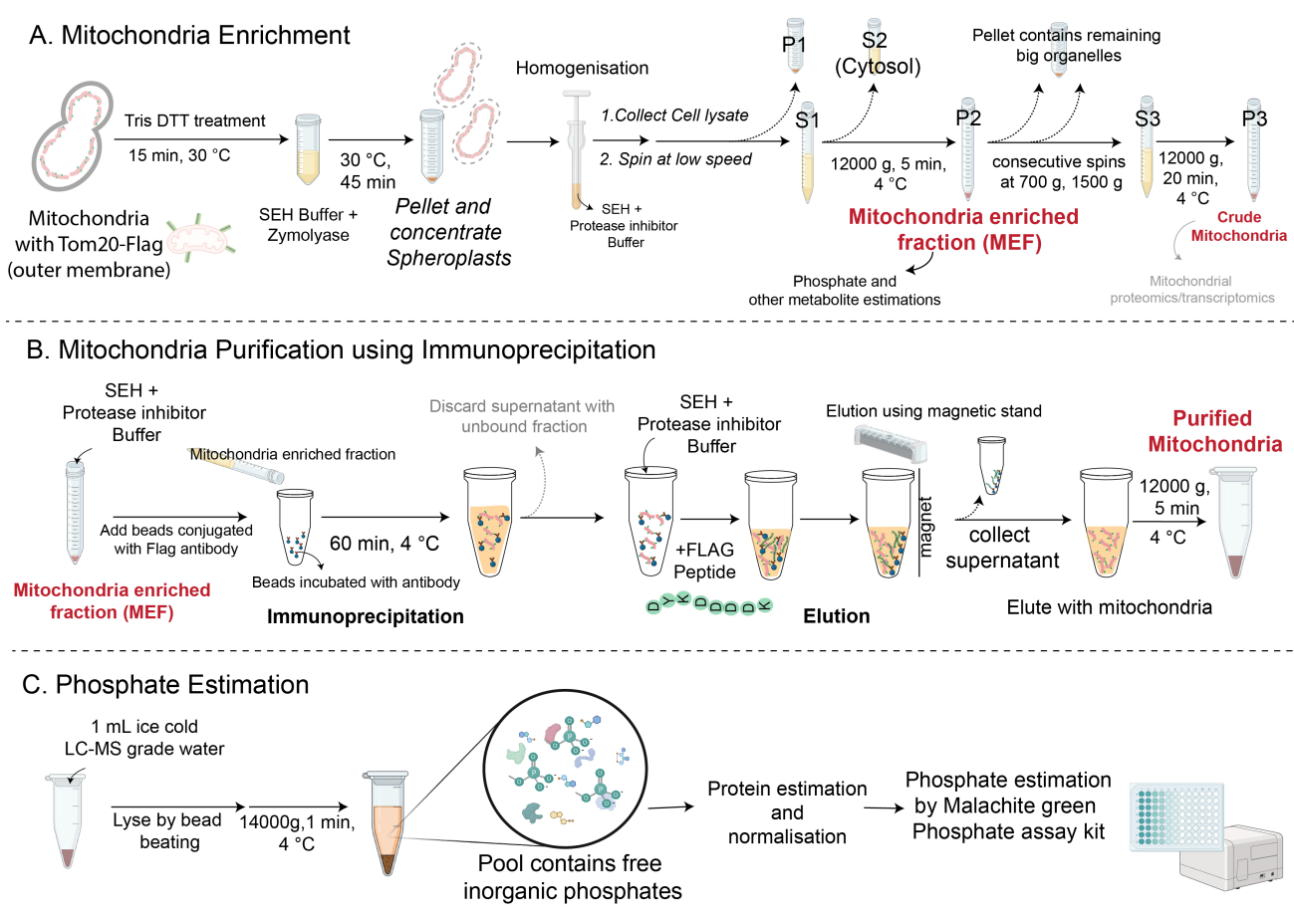


## Background

Mitochondria are essential for cellular energetics and ATP production via oxidative phosphorylation [1]. To study mitochondrial function, isolated mitochondria are commonly used and obtained through techniques such as differential centrifugation and pull-down protocols [2–10]. These methods ensure mitochondrial enrichment while preserving their integrity for various activities. However, there is no standardized method for extracting metabolites from isolated mitochondria, particularly from model cells like yeast. Aiming to address this gap, our protocol, which was used extensively in Vengayil et al. [11], provides a reliable method to extract and quantitatively measure free inorganic phosphate (Pi) from both isolated mitochondria and the cytosol. This approach enables precise metabolite analysis and compares relative cytosolic and mitochondrial pools of a metabolite, expanding the scope of mitochondrial research.

Some advantages of the described protocol for extracting and quantitatively measuring free inorganic phosphates are as follows: 1) Specificity for inorganic phosphates: Coupling a phosphate estimation method with a purified mitochondrial fraction ensures a compartment-based analysis of phosphate measurements without interference from other cellular compartments. For example, vacuoles have inorganic phosphates and, therefore, will require a different linear range for measurements. 2) The existing protocol is applicable for phosphate measurements in both mitochondrial and cytosolic compartments and can extend to other metabolites. 3) Reproducibility: This protocol for mitochondrial isolation and phosphate measurements is optimized for consistency and reproducibility. 4) Future scope: This protocol can be modified and coupled with metabolomics or proteomics to get comprehensive data on mitochondrial metabolite compositions.

## Materials and reagents

**Biological materials**

*1. Saccharomyces cerevisiae* (CEN.PK background [12]), with FLAG-tagged Tom20 (Tom20 protein with a 3×-FLAG epitope tag included at the endogenous C-terminal locus) [11]

**Reagents**

1. Yeast extract (Gibco Bacto, catalog number: 212750)

2. Peptone (Gibco Bacto, catalog number: 211677)

3. Glucose (Sigma-Aldrich, CAS number: 50-99-7)

4. Sorbitol (Sigma-Aldrich, catalog number: S6021, CAS number: 50-70-4)

5. Potassium dihydrogen phosphate (Sigma-Aldrich, CAS number: 7778-77-0)

6. Tris (hydroxymethyl) aminomethane (Tris base) (Sigma-Aldrich, CAS number: 77-86-1)

7. DTT (1,4-Dithiothreitol) (Sigma-Aldrich, catalog number: D0632, CAS number: 3483-12-3)

8. HEPES buffer [4-(2-hydroxyethyl) piperazine-1-ethane-sulfonic acid] (HIMEDIA, catalog number: RM380, CAS number: 7365-45-9)

9. Potassium hydroxide (KOH) (Sigma-Aldrich, CAS number: 1310-58-3)

10. Magnesium chloride (Sigma-Aldrich, CAS number: 7791-18-6)

11. Zymolyase-20T (MP Biomedical, catalog number: 08320921)

12. Pepstatin (Sigma-Aldrich, catalog number: P5318)

13. Leupeptin hemisulfate (Selleck Chemicals, catalog number: S7380)

14. EDTA (Sigma-Aldrich, CAS number: 6381-92-6)

15. Anti-FLAG mouse antibody (Sigma-Aldrich, catalog number: F1804)

16. FLAG peptide (DYKDDDDK) (Sigma-Aldrich, catalog number: F3290)

17. HPLC-grade water (Fisher Chemical, Water, Optima LC-MS Grade, CAS number: 7732-18-5)

18. Malachite Green Phosphate Assay kit (Cayman chemicals, catalog number: 10009325)

19. BCA Protein Estimation kit (G-biosciences, catalog number: 786-570)

20. Sulfuric acid (H_2_SO_4_) (Fischer scientific, catalog number: Q29995)

**Solutions**

1. Tris-DTT buffer (see Recipes)

2. Sorbitol solution (see Recipes)

3. HEPES-KOH solution (see Recipes)

4. SEH buffer (see Recipes)

5. SEH buffer + protease inhibitors (see Recipes)

6. Zymolyase solution (see Recipes)

7. Media (see Recipes)

a. 50% Glucose

b. Glucose [YPD (yeast extract, peptone, dextrose)] media

c. Ethanol [YPE (yeast extract, peptone, ethanol)] media

**Recipes**

**1. Tris-DTT buffer**

**Table:** 

Reagent	Final concentration	Quantity or volume
1 M Tris-H_2_SO_4_ pH 9.4	100 mM	5 mL
1 M DTT	10 mM	0.5 mL
Autoclaved MilliQ water	N/A	44.5 mL
Total	N/A	50 mL

*Note: For 1 M Tris-H_2_SO_4_ buffer, weigh 12.11 g of Tris base and dissolve in 80 mL of MilliQ water. Adjust the pH to 9.4 with H_2_SO_4_ (volume as required for the adjustment). After pH adjustment, make the volume to 100 mL with water. Store the Tris-H_2_SO_4_ buffer stock at room temperature. Store the DTT stock at -20 °C. Prepare the Tris-DTT buffer fresh and prewarm to 30 °C for 15 min before use.*

**2. Sorbitol solution**

**Table:** 

Reagent	Final concentration	Quantity or volume
Sorbitol	2.4 M	87.4 g
Autoclaved MilliQ water	N/A	200 mL
Total	N/A	200 mL

The 2.4 M stock solution of sorbitol is made in ddH_2_O and autoclaved. The solution can be stored at room temperature for 6 months or until contaminated.

Start by dissolving 87.4 g of sorbitol in 90 mL of water and make up the volume in the end, when all the powder is dissolved.

**3. HEPES-KOH solution**

**Table:** 

Reagent	Final concentration	Quantity or volume
HEPES	1 M	11.915 g
KOH	N/A	to pH 7.4
Autoclaved MilliQ water	N/A	50 mL
Total	N/A	50 mL

For the 1 M stock solution of HEPES, adjust pH to 7.4 with KOH and make up the final volume to 50 mL with water. Autoclave the solution and store at room temperature for up to a year.

**4. SEH buffer**

**Table:** 

Reagent	Final concentration	Quantity or volume
2.4 M sorbitol	0.6 M	75 mL
1 M HEPES-KOH	20 mM	6 mL
Autoclaved MilliQ water	N/A	219 mL
Total	N/A	300 mL

Always use a freshly prepared SEH buffer for each experiment. Optional: Add 1 mM EDTA to the buffer to reduce enzyme activity.

**5. SEH buffer + protease inhibitors**

**Table:** 

Reagent	Final concentration	Quantity or volume
2.4 M Sorbitol	0.6 M	75 mL
1 M HEPES-KOH	20 mM	6 mL
1.45 mM pepstatin	1 μM	0.207 mL
50 mM Leupeptin	100 μM	0.6 mL
Autoclaved MilliQ water	N/A	218.2 mL
Total	N/A	300 mL

The pepstatin stock can be prepared in ethanol by heating to 60 °C to dissolve it.

Any standard protease inhibitor cocktail compatible with immunoprecipitations can be used here. Make this buffer right before use.

Optional: If preferred, 1 mM phenylmethylsulfonyl fluoride (PMSF) can be added, but it may interfere with protein estimation methods.

**6. Zymolyase solution**

**Table:** 

Reagent	Final concentration	Quantity or volume
Zymolyase	25 mg/g cell pellet	25 mg
SEH buffer	5 mL	5 mL
Total	N/A	5 mL

Freshly prepare zymolyase solution in SEH buffer. The final calculation depends on the pellet volume measured in step A2 (see below). The table above has calculations for 1 g of pellet size.

**7. Media**

**a. 50% glucose:** Make 50% solution (w/v) of glucose in water and filter sterilize. This is the glucose stock solution for preparing YPD media.

**b. Glucose (YPD) media:** For the YPD solution, add 1 g (1%) of yeast extract and 2 g (2%) of peptone to 100 mL of water (YP solution) and autoclave. Then, add 2% glucose from the sterilized stock solution (50% glucose) to the desired volume. For 100 mL of YPD, make a solution of 96 mL of YP and 4 mL of 50% glucose.

**c. Ethanol (YPE) media:** After making the YP solution and autoclaving, add 2% ethanol to make the YPE media. For 100 mL of YPE, add 2 mL of 100% ethanol to 98 mL of YP solution.

**Laboratory supplies**

1. Erlenmeyer (conical) flasks (Borosil Scientific, catalog numbers: 4980016 and 4980012)

2. Pipettes (Eppendorf India, catalog number: 05-403-151)

3. Spinwin tube conical bottom (Tarsons, catalog numbers: 500031 and 500041)

4. Spinwin microcentrifuge tubes (Tarsons, catalog numbers: 500010 and 500020)

5. Kimble Dounce tissue grinder set (Sigma-Aldrich, catalog number: D8938)

6. 96-well plates (SPL Life Sciences, catalog number: 30096)

7. Dynabeads^TM^ Protein G (Invitrogen, catalog number: 10003D)

## Equipment

1. Weighing balance (aczet, model: CY 3102)

2. Centrifuge (Eppendorf, model: centrifuge 5424 R, 5910 R)

3. Heating block (IKA Dry Block Heater 1, catalog number: 0004025100)

4. Cyclomixer (IKA Loopster, catalog number: 0004015000)

5. DynaMag^TM^-2 magnetic stand (Invitrogen, catalog number: 12321D)

6. Shaker and incubator (Scigenics Biotech Orbitek (model: Orbitek LT-D) and Sanyo Incubator (model: MIR-262)

7. Plate reader (Thermo Fisher Scientific, model: Multimode Microplate Reader, Varioskan LUX)

## Software and datasets

1. GraphPad Prism 9.0.1 was used for generating plots and performing statistical analysis. Other software such as R Studio can also be used

## Procedure

**A. Preparation of mitochondria-enriched fraction and crude mitochondria**

1. Yeast growth conditions: Inoculate the *S. cerevisiae* strain in 25 mL of YPD media (see Recipes) overnight (16 h) at 30 °C in a shaking incubator at 250 rpm. Use this primary culture to start a secondary culture to grow yeast cells (tagged with Tom20) to mid-log phase (0.6~0.8 OD_600_) before harvesting.

*Note: The volume of the secondary culture depends on the media used and subsequent processing. In our study, we use 150 mL for ethanol media and 300 mL for the glucose media (see Recipes).*

2. Collect cells by centrifugation at 1,500× *g* for 5 min at 4 °C, remove the supernatant, and wash the pellet by resuspending in ice-cold water. Centrifuge the resuspended cell pellet at 1,500× *g* for 5 min at 4 °C.

*Notes:*

*1. Prepare all the necessary buffers before starting this step.*

*2. Weigh the cell pellet (wet weight). To determine the wet weight, first weigh an empty tube and tare the balance. Then, place the tube containing the washed pellet and weigh it again. This is important for determining the volume of solutions used in subsequent steps. It is not recommended to freeze and store the pellet since freezing can rupture organelles, which might affect the efficiency of the intact mitochondria isolation.*

3. Incubate the cells in Tris-DTT buffer (5 mL/g wet weight) for 15 min at 30 °C with shaking at 250 rpm. (Prewarm the Tris-DTT buffer at 30 °C.)

*Note: Prepare the zymolyase solution (25 mg/g yeast wet weight) during this incubation.*

4. Wash the pellet with SEH buffer (5 mL/g wet weight) and centrifuge at 1,500× *g* for 5 min at 4 °C.

*Note: Several mitochondrial isolation protocols use SP [1.2 M sorbitol and 20 mM potassium phosphate (KPi) solution)] buffer along with the SEH buffer. If mitochondria are being used for Pi estimation, SP buffer is not recommended since it contains potassium phosphate, which interferes with final Pi estimations.*

5. Incubate with zymolyase solution (resuspended in 25 mg of zymolyase per gram of yeast wet weight in 5 mL of SEH buffer) at 30 °C, shaking (250 rpm) for 45 min to 1 h to produce spheroplasts.

6. Centrifuge at 4,500× *g* for 5 min to concentrate spheroplasts, wash with ice-cold SEH buffer (5 mL/g yeast wet weight), and resuspend in ice-cold SEH buffer with protease inhibitor cocktail (5 mL/g yeast wet weight).

*Note: If the maximum speed of the centrifuge is <4,500*× *g, increase the time of the spin to 10 min or more.*

7. Homogenize with 15 forceful strokes (about 1–1.5 min per sample) using a pre-chilled glass Dounce tissue grinder on ice.

*Note: Extra volume in the apparatus can lead to spillage and loss of sample. It is advisable to fill less than half of the tube.*

8. Centrifuge the homogenized samples at 1,500× *g* for 5 min at 4 °C. Collect the pellet P1 and supernatant S1. P1 contains the cell membrane, vacuole, and other debris; S1 contains the cytosol, mitochondria, and ER.

9. Spin the supernatant S1 at high speed (12,000× *g*) for 5 min at 4 °C and collect the pellet P2 and supernatant S2. P2 contains mitochondria (and ER and cytosol components too), and S2 contains cytosol. Weigh the pellet P2.

*Note:* ***P2 is the mitochondria-enriched fraction (MEF).*** *This pellet can be used to immunoprecipitate mitochondria for phosphate and metabolite measurements. For isolating mitochondria by immunoprecipitation, proceed to step B1. To obtain the crude mitochondrial fraction, proceed to step A10. Steps A10–A12 can be followed if the aim is to achieve a more refined mitochondria for mitochondrial proteomics. However, for phosphate and other metabolite estimations, immunoprecipitation is recommended to obtain pure mitochondria, since vacuolar and ER contamination in the crude mitochondrial fraction can interfere with the metabolite estimations.*

10. Resuspend P2 in ice-cold SEH buffer containing protease inhibitors (1 mL/g yeast wet weight).

11. Centrifuge the suspension from step A9 at 700× *g* for 5 min, collect the supernatant (not shown in the graphical abstract), and spin again at 1,500× *g* for 5 min at 4 °C. This is to remove any remaining big organelles. Collect the supernatant S3.

12. Spin the supernatant S3 at high speed (12,000× *g*) for 10 min at 4 °C. Collect the pellet P3 containing mitochondria. Note the weight of the pellet.

13. Resuspend P3 in ice-cold SEH buffer containing protease inhibitors (1 mL/g wet weight). This is the **crude mitochondria fraction** and contains some contamination from the ER and cytosol.

*Note: This fraction has mostly purified mitochondria and reduced contamination compared to P2. This could be used for isolating mitochondrial proteins and activity assays. However, for phosphate and metabolite estimation, mitochondria are further purified by immunoprecipitation. For this, P2 (step A9) can be used.*

**B. Purification of mitochondria using immunoprecipitation**

**Continued from step A9**

*Note: Use cells with TOM20-FLAG for these steps.*

1. Resuspend the pellet P2 in 700 μL of ice-cold SEH with protease inhibitors.

*Note: Steps B1–B6 are performed in a cold room.*

2. Aliquot 1.5 mg Dynabeads protein G (50 μL from 30 mg/mL stock) in a 1.5 mL microcentrifuge tube. Place the tube in the magnetic separation rack for 30 s.

3. Aspirate the liquid with a micropipette and wash the beads with 100 μL of SEH buffer.

4. Resuspend the Dynabeads in 100 μL of SEH buffer and add 10 μg of anti-FLAG antibody. Incubate the Dynabeads–antibody mixture for 15 min with gentle rotation (15 rpm) on a cyclomixer.

5. Add the resuspended mitochondrial fraction (from step B1) to antibody-conjugated Dynabeads, mix, and incubate for 30–60 min with gentle rotation (15 rpm).

6. Place the beads in the magnetic rack for 1 min and collect the supernatant (this is the flowthrough that contains unbound mitochondria).

7. Wash the beads twice with 300 μL of ice-cold SEH buffer.

8. Elution: Resuspend the beads in 10 μL of SEH buffer + 30 μL of FLAG peptide (from a 2 mg/mL stock, final concentration 1.5 mg/mL) and incubate at room temperature for 30 min with intermittent mixing to elute the mitochondria.

9. Place the suspension in the magnetic rack for 1–2 min and collect the supernatant containing the purified mitochondria into a fresh 1.5 mL microcentrifuge tube.

10. Centrifuge the supernatant from step B9 at 12,000× *g* for 5 min. The pellet contains purified mitochondria.

*Note: This protocol can also be used to obtain enriched cytosol (S2) and purified mitochondria (step A9 of immunoprecipitation). To obtain purified cytoplasmic and vacuolar extracts, refer to Schwencke et al [10].*

**C. Phosphate extraction and estimation**

1. Resuspend the pellet from step B10 containing purified mitochondria in 1 mL of ice-cold LC–MS-grade water.

*Note: LC–MS-grade water is recommended to avoid any phosphate impurities in water that might affect the assay readout.*

2. Lyse the resuspended mitochondria by bead beating (add glass beads corresponding to a volume of 100 μL, followed by bead beating for 3 × 20 s), with intermittent cooling for 30–60 s between each bead beating.

**Pause point:** The lysates can be collected and stored at -80 °C for western blotting.

3. Centrifuge the lysates at 20,000× *g* (14,000 rpm) for 1 min at 4 °C and collect 700–800 μL of supernatant. The supernatant contains inorganic phosphate.

*Note: Collect equal amounts of supernatants between samples for more consistency. Free phosphates can be extracted in water. Other metabolites can be extracted from this mitochondrial pellet using ethanol using the method from Walvekar et al. [13]*

**Pause point:** The supernatants can be stored at 4 °C for Pi and protein estimations. Perform protein estimation before starting the assay.

4. Estimate the protein amounts in the supernatant using a BCA Protein Estimation kit. This protein amount can be used to normalize the Pi measurements.

*Note: Some reagents, like PMSF and DTT, can interfere with BCA estimation. In this protocol, we use a low concentration of DTT at the beginning, which is removed in subsequent steps, so it should not interfere.*

5. Estimate Pi using the Malachite Green Phosphate Assay kit (see Section D). Create a standard curve using Pi standards provided in the kit and estimate the Pi amounts in the supernatant from step C3. It is recommended to use multiple dilutions [undiluted or diluted in LC–MS-grade water (1:5, 1:10, or any suitable dilutions)] to ensure that the assay readouts are in the range of the phosphate standards used.

**D. Malachite green assay**

Malachite green assay measures free phosphate by estimating molybdophosphoric acid formed by the reaction of malachite green molybdate and free phosphate under acidic conditions. The green molybdophosphoric acid complex formed in the reaction is measured at 620–640 nm, providing a readout for the free phosphate concentrations. Any standard malachite green assay kit can be used to estimate mitochondrial phosphate concentrations. In this protocol, the Malachite Green Assay kit from Cayman Chemicals was used.

The detailed protocol for performing phosphate estimation by malachite green assay is provided on the manufacturer’s website (https://www.caymanchem.com/product/10009325). This protocol is suitable for use with the mitochondrial lysate obtained from step C3. Phosphate standards are prepared, and Pi levels are estimated in the samples according to the manufacturer’s instructions.

1. Dilute the 1 M phosphate standard provided in the kit using LC–MS-grade water. Serially dilute this phosphate standard in 1.5 mL tubes to generate concentrations of 12.5 μM, 6.25 μM, 3.13 μM, and1.56 μM. LC–MS-grade water is used as a blank standard (0 μM). Aliquot 50 μL of phosphate standards and the blank into the wells of a 96-well plate.

2. Dilute the supernatant from step C3 (mitochondrial lysate containing phosphate) at 1:5 and 1:10 dilutions using LC–MS-grade water to a final volume of 100 μL.

3. Aliquot 50 μL of the diluted samples into the wells of a 96-well plate.

*Note: Diluting the samples is recommended since the undiluted supernatant from step C3 might contain phosphate concentrations that might fall outside of the linear range of the assay. If you are trying the protocol for the first time, it is recommended to use undiluted samples and multiple sample dilutions to identify the best dilution suitable for the assay.*

**Figure 1.**
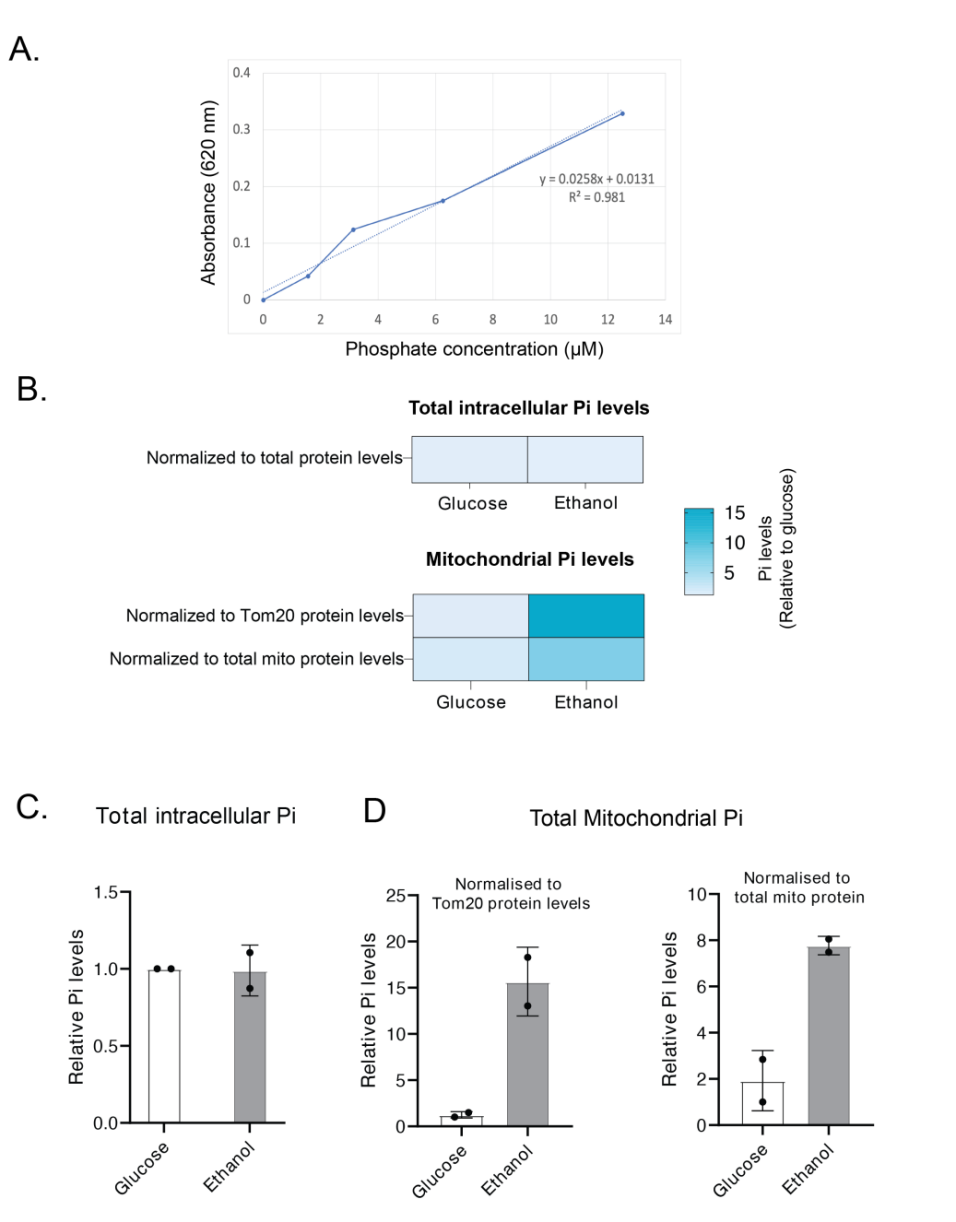
Total intracellular and mitochondrial phosphate estimation from *S. cerevisiae* cells grown in ethanol and glucose medium. Cells were grown in log phase to an OD_600_~0.8, and total Pi levels (from whole cells) or mitochondrial Pi levels (from isolated mitochondria) were estimated by malachite green assay. (A) A phosphate standard curve was plotted using different concentrations of phosphate standards. The Pi levels in isolated mitochondria were estimated and plotted relative to Pi levels in glucose-grown cells. Total Pi levels were normalized to total protein levels, and mitochondrial Pi levels were normalized to Tom20 or total mitochondrial protein levels. (B) A heat map representation comparing total and mitochondrial Pi levels in glucose- vs. ethanol-grown cells. (C, D) Quantitative representation of panel B. Data displayed as mean ± SD, n = 2 independent biological replicates.

4. Add 5 μL of MG acidic solution (included in the kit) to each well containing samples or standards, mix by gentle tapping, and incubate the plate at room temperature for 10 min.

5. Add 15 μL of MG Blue solution (included in the kit) to each well containing samples and standards, mix by gentle tapping, and incubate the plate at room temperature for 20 min.

6. Measure the absorbance in each well at 620 nm using a microplate reader.

7. The Pi measurements can be normalized to total protein in the samples from step C3. Additionally, the resuspended mitochondria from step B10 can be used to estimate the Tom20 or Idh1 protein levels by western blotting. The Pi levels can be normalized to Tom20, Idh1, or any suitable mitochondrial protein (Figure 1C).

*Note: Normalizing Pi levels to Tom20, Idh1, or any single mitochondrial protein is* ***not recommended*** *when comparing samples grown in different media. Since growth conditions can influence the expression of individual proteins, it is recommended to normalize Pi levels to total protein to ensure accurate comparisons (Figure 1C). In case of normalization to a single protein level, western blots have to be performed to confirm that the total level of this protein is constant across conditions where Pi is being compared.*

## Data analysis

Pi levels were estimated by plotting a standard curve using the phosphate standards provided in the Pi Estimation Assay kit (Figure 1A). The best-fitting line was plotted using Microsoft Excel. Pi amounts in the samples were estimated from this standard curve by solving for the equation y = mx + c, where y is the absorbance, m is the slope, x is the Pi amount, and c is the y-intercept. Once Pi amounts in samples were estimated, statistical analysis was performed using an unpaired Student’s t-test in GraphPad Prism 9.0.1. There were no significant differences in total phosphate levels in cells grown in glucose and ethanol media. However, mitochondrial phosphate levels were significantly higher in ethanol-grown cells than in glucose-grown cells (Figure 1). Therefore, our protocol can be used for measuring intracellular phosphate pools in different cellular compartments.

## Validation of protocol

This protocol has been used and validated in the following research article:

- Vengayil et al. [11]. The deubiquitinase Ubp3/Usp10 constrains glucose-mediated mitochondrial repression via phosphate budgeting. *eLife* (Figure 4B, Figure 4, supplement 1A).

Three biological replicates were used in these experiments; the data provided in Figure 1 in this protocol are from two biological replicates.

## General notes and troubleshooting

**General notes**

1. It is recommended to perform western blots for mitochondrial, cytosolic, and vacuolar proteins in different fractions of the isolation procedure to ensure efficient mitochondrial purification.

2. The isolated mitochondria pellets can be resuspended in appropriate solutions depending on the experiment. For example, in the case of metabolomics, the pellet can be resuspended in 60% methanol, and for proteomics applications, the pellet can be resuspended in a Tris-SDS buffer.

3. If required, this method of mitochondrial isolation can be followed by native PAGE to determine mitochondrial respiratory chain activity.

4. For proteomics or native PAGE analysis of mitochondrial proteins, a crude mitochondrial fraction can also be used.

5. This protocol is adapted to be used for yeast mitochondrial isolation, but similar protocols are used for mammalian cells [3,6,8].

**Troubleshooting**

**Problem 1:** No purified mitochondrial pellet after immunoprecipitation.

Possible causes: 1. Insufficient cells. 2. Low mitochondrial yield in the mitochondria-enriched fraction.

Solution: 1. Increase the volume of the secondary culture. 2. Confirm mitochondrial enrichment by western blotting for different fractions obtained during isolation.

**Problem 2:** No detectable phosphate in the immunopurified mitochondria.

Possible cause: Phosphate amount in the sample is below the detection limit.

Solution: Ensure that the immunopurified fraction contains mitochondria and increase the volume of the sample used in the Pi assay.

*Note: It is advisable to start with more starting material to avoid issues related to loss.*

**Problem 3:** No mitochondrial pool detected in the elution.

Possible causes: 1. Sample is lost in the flowthrough. 2. Sample remains stuck to the beads.

Solution: Run all the fractions collected during immunoprecipitation to note all the steps where loss is occurring. Increase the binding time (step B5) to more than 1 h to ensure mitochondrial binding to the beads. Increase the concentration of Flag peptide for the elution (step B8) and incubate longer.

**Problem 4:** Interference in BCA estimation.

Possible cause: PMSF, DTT, TCEP, or other buffer ions that can interfere with BCA reagents.

Solution: It is recommended to avoid using these in mitochondrial isolation. However, if necessary, a) dilute the samples to dilute the interfering compound for BCA estimation, and b) use the same concentration of interfering reagent (usually buffer used) as a blank to nullify any signal coming from interference.
